# Novel Pathways Revealed in Bursa of Fabricius Transcriptome in Response to Extraintestinal Pathogenic *Escherichia coli* (ExPEC) Infection

**DOI:** 10.1371/journal.pone.0142570

**Published:** 2015-11-10

**Authors:** Hongyan Sun, Peng Liu, Lisa K. Nolan, Susan J. Lamont

**Affiliations:** 1 Department of Animal Science, Iowa State University, Ames, Iowa, 50011, United States of America; 2 Department of Statistics, Iowa State University, Ames, Iowa, 50011, United States of America; 3 Department of Veterinary Microbiology and Preventive Medicine, Iowa State University, Ames, Iowa, 50011, United States of America; University of Münster, GERMANY

## Abstract

Extraintestinal pathogenic *Escherichia coli* (ExPEC) has major negative impacts on human and animal health. Recent research suggests food-borne links between human and animal ExPEC diseases with particular concern for poultry contaminated with avian pathogenic *E*. *coli* (APEC), the avian ExPEC. APEC is also a very important animal pathogen, causing colibacillosis, one of the world’s most widespread bacterial diseases of poultry. Previous studies showed marked atrophy and lymphocytes depletion in the bursa during APEC infection. Thus, a more comprehensive understanding of the avian bursa response to APEC infection will facilitate genetic selection for disease resistance. Four-week-old commercial male broiler chickens were infected with APEC O1 or given saline as a control. Bursas were collected at 1 and 5 days post-infection (dpi). Based on lesion scores of liver, pericardium and air sacs, infected birds were classified as having mild or severe pathology, representing resistant and susceptible phenotypes, respectively. Twenty-two individual bursa RNA libraries were sequenced, each yielding an average of 27 million single-end, 100-bp reads. There were 2469 novel genes in the total of 16,603 detected. Large numbers of significantly differentially expressed (DE) genes were detected when comparing susceptible and resistant birds at 5 dpi, susceptible and non-infected birds at 5 dpi, and susceptible birds at 5 dpi and 1 dpi. The DE genes were associated with signal transduction, the immune response, cell growth and cell death pathways. These data provide considerable insight into potential mechanisms of resistance to ExPEC infection, thus paving the way to develop strategies for ExPEC prevention and treatment, as well as enhancing innate resistance by genetic selection in animals.

## Introduction

Avian pathogenic *Escherichia coli* (APEC), a subpathotype of the extraintestinal pathogenic *E*. *coli* (ExPEC) pathotype, can infect many avian species (chickens, turkeys, and ducks) worldwide as a primary or secondary pathogen [1]. Three predominant serogroups, APEC O1, O2, and O78, account for 15–60% of total APEC isolates [2, 3]. Syndromes caused by APEC include localized or systemic infections, collectively known as colibacillosis [4]. Recent findings demonstrate that there are significant genetic similarities and disease-causing traits and abilities between APEC and human ExPEC [5–7] and that APEC-like organisms may contaminate retail poultry meat [8]. Altogether, these findings suggest that some APEC are capable of causing such human diseases as urinary tract infections, sepsis and neonatal meningitis following ingestion or handling contaminated poultry products [8–10]. Control of APEC, therefore, is highly desirable for reasons of both animal and human health.

Antimicrobial drugs were extensively utilized in the past in poultry to treat, prevent and control colibacillosis. However, consumers are expressing increasing concerns about antimicrobial use in animal production, and drugs available to producers, such as fluoroquinolones, are increasingly restricted, forbidden or scrutinized for use in poultry production. Additionally, multi-antibiotic resistant bacteria are emerging [11]. Vaccination, too, has its limitations, with many providing only serotype-specific protection against APEC [12, 13]. Consequently, genetic selection for birds that are innately resistant against APEC presents a more efficient and permanent way to control APEC infection. To accomplish this goal, however, a better understanding of host immunological responses and genetic resistance mechanisms are needed.

The Bursa of Fabricius, a major site for B cell proliferation and diversification, is a unique immune tissue of birds compared to mammals [14, 15]. The primary function of the bursa is to provide the environment in which bursal cells undergo rearrangement of the immunoglobulin gene V(D)J segments to generate B cell receptors and mature B cells [14–16]. Thus, the Bursa of Fabricius has a major role in normal development of avian B cell lineage specification and commitment and, therefore, a major role in effective antibody response in host defense. IgY can be effective in defense against colibacillosis [17] and *Clostridium perfringens* infection [18]. IgY- and IgM- containing plasma cells abundantly occurred in bursa in broiler chickens that were vaccinated with *Newcastle* disease virus (NDV) vaccine [19]. Also, marked atrophy of bursa was observed in natural colibacillosis of broiler chickens [20] and the relative weights of bursa was dramatically decreased at 1 day post-inoculation [21]. B lymphocytes were greatly depleted in bursa after 1 day post-infection in colibacillosis of white leghorn, as assessed by histology [21]. The crucial functions of the bursa in colibacillosis, therefore, led us to characterize the bursa transcriptome in response to APEC infection to help design better strategies to control APEC.

Moreover, the chicken is a unique model organism in contrast to mammals, in that birds possess a bursa of Fabricius, enabling detailed study of maturing B cell activity during APEC infection, which offers useful insights into ExPEC pathogenesis. The aim of the study was to identify genes and pathways that are differently expressed in susceptible versus resistant chickens to aid our understanding of the genetic control of APEC pathology.

## Materials and Methods

### Ethics statement

All animal care and experimental procedures were reviewed and approved by the Institutional Animal Care and Use Committee of Iowa State University (Log #11-07-6460-G).

### Animal study

**Fig 1:**
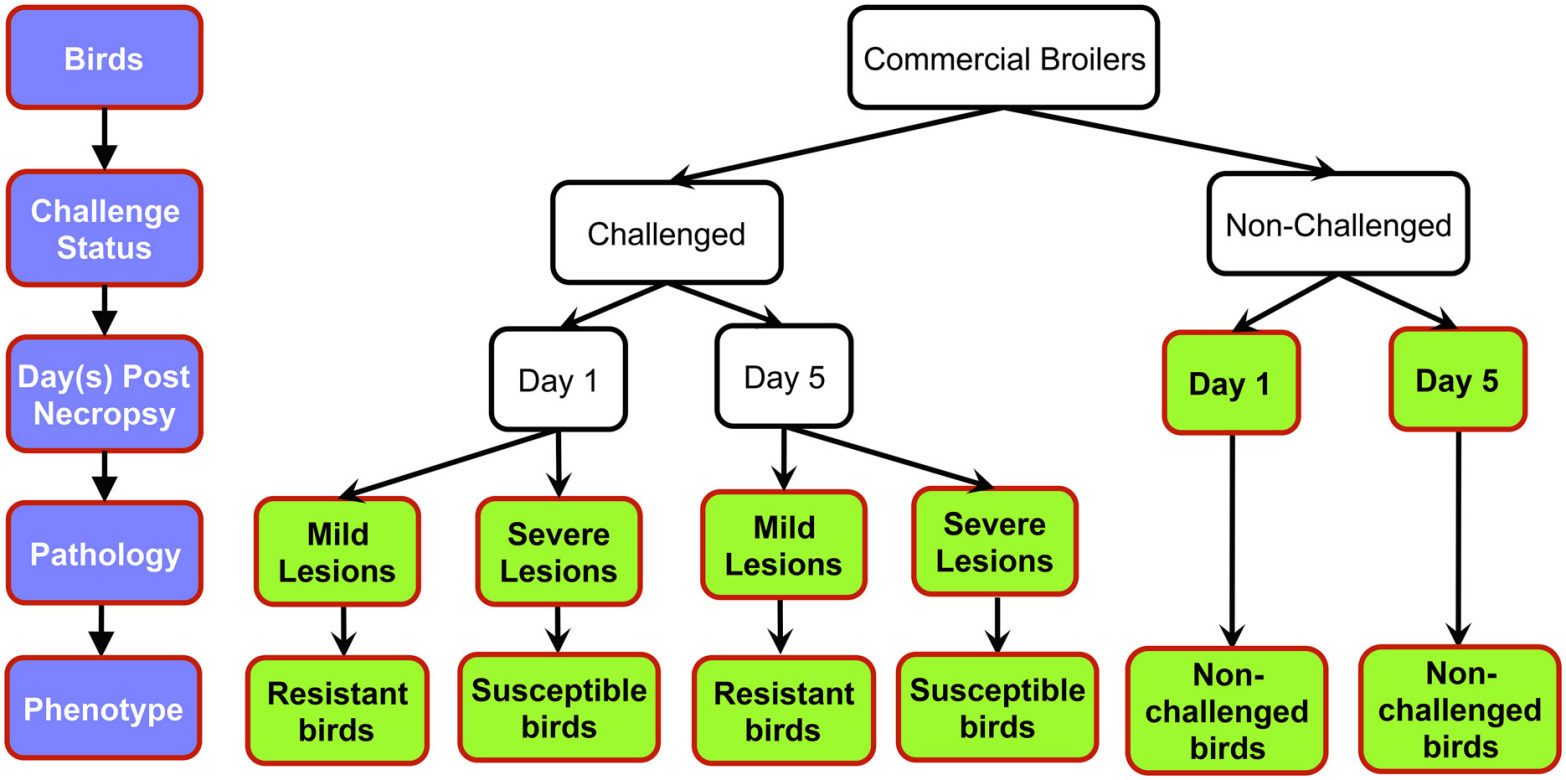
Experimental Design. Six treatments were generated by challenge status, necropsy time post-infection and pathology level of challenged birds: non-challenged birds at 1 or 5 days, challenged resistant birds at 1 or 5 days post-infection (dpi), and challenged susceptible birds at 1 or 5 dpi.

In this RNAseq study, we used four birds each for susceptible and resistant groups at 1 dpi and 5 dpi, and three birds each for non-challenged groups at 1 dpi and 5 dpi, totalling 22 samples. At four weeks of age, 288 commercial broiler male birds were challenged with 0.1 ml APEC O1 (10^8^ colony-forming units) into the left thoracic air sac. This large number was used to enable a clear separation of resistant from susceptible phenotypes. Control birds were injected with 0.1 ml of phosphate buffered saline (PBS) via the same route. Bursas were harvested at 1 and 5 days post-infection (dpi). For the sample collection times, the early day (1 dpi) was to assess the immediate response to infection [22] while 5 dpi was the time of maximal symptoms [5]. Based upon necropsy-scored lesions on the liver, pericardium, and air sacs, the challenged birds were assigned to mild or severe pathology categories, representing resistant and susceptible phenotypes, respectively. The lesion scores for the liver, pericardium, and air sacs were 0–2, 0–2, and 0–3, respectively, as described by Peighambari et al. [23]. Birds were categorized as resistant (mild lesions) if the sum of the three lesion scores for that individual totaled 0 to 2; birds were categorized as susceptible (severe lesion) if the lesion scores totaled 5 to 7. Table 1 displays the lesion score and body weight information for each bird of this RNAseq study. Six treatment groups, therefore, were generated: non-challenged birds at 1 or 5 dpi, challenged-resistant birds at 1 or 5 dpi, and challenged-susceptible birds at 1 or 5 dpi (Fig 1). Detailed information on the APEC O1 strain and experimental procedures was previously published [24, 25].

**Table 1:** Lesion Scores, Pathology Class and Phenotype of Bird for Each Sample.

Phenotype	Collection day (s)	Bird number	Air sac scores	Pericardium scores	Liver scores	Total scores	Pathology class	Body weight (kg)
Non-challenged	1	1	0	0	0	0	None	1.05
Non-challenged	1	2	0	0	0	0	None	0.95
Non-challenged	1	3	0	0	0	0	None	1.10
Non-challenged	5	1	0	0	0	0	None	1.80
Non-challenged	5	2	0	0	0	0	None	1.75
Non-challenged	5	3	0	0	0	0	None	1.87
Resistant	1	1	1	1	0	2	Mild	1.00
Resistant	1	2	0	0	0	0	None	1.05
Resistant	1	3	1	0	0	1	Mild	1.00
Resistant	1	4	1	0	0	1	Mild	1.10
Resistant	5	1	0	1	0	1	Mild	1.75
Resistant	5	2	0	0	0	0	None	1.67
Resistant	5	3	0	0	0	0	None	1.70
Resistant	5	4	1	1	0	2	Mild	1.65
Susceptible	1	1	2	2	1	5	Severe	1.00
Susceptible	1	2	3	2	1	6	Severe	1.05
Susceptible	1	3	2	2	1	5	Severe	1.00
Susceptible	1	4	2	2	2	6	Severe	0.95
Susceptible	5	1	3	2	2	7	Severe	1.15
Susceptible	5	2	3	2	2	7	Severe	1.22
Susceptible	5	3	3	2	2	7	Severe	1.30
Susceptible	5	4	3	2	2	7	Severe	1.20

Note: dpi, day post-infection.

### RNA Extraction, cDNA Library Preparation, and RNA sequencing

Bursas stored in RNAlater were subjected to total RNA extraction using the Ambion MagMAX-96 Kit (AM1839) (Applied Biosystems, Foster City, CA) according to the manufacturer’s directions. To assess the quality and quantity of RNA, the Agilent 2100 Bioanalyser (Agilent Technologies) was used to generate an RNA Integrity Number (RIN). The RIN score of all RNA samples was greater than 8.0. Total RNA (0.1–4 μg) was used for library preparation. Twenty-two RNA samples (4 per each challenged group, and 3 per each non-challenged group) were processed to produce 22 individual cDNA libraries using the Illumina TruSeq ^®^ RNA Sample Preparation v2 Kit per the manufacturer’s instructions (Protocol: #15026495, May 2012). Libraries were quantitated using the Qubit^®^ Quantitation Platform and HS dsDNA kit (Invitrogen, Paisley, UK). To control for lane and batch effects, each lane contained a sample from each of the six treatment groups. Four lanes of an Illumina^®^ HiSeq 2000 instrument at DNA facility in Iowa State University (ISU) were used to generate a total of 537.2 million 100bp single-end reads. Demultiplexed fastq files were generated using Illumina CASAVA software.

### Reads Quality Control, Alignment, and HTseq-count

Quality control of reads was implemented through FastQC software (version 0.10.1) with a Phred score of 32. Fastx toolkit software (http://hannonlab.cshl.edu/fastx_toolkit/) was used to trim the adaptor. Then, sequencing reads were aligned to the Ensemble Gallus gallus 4.0 reference genome through the TopHat software (version 2.0.9) and Bowtie (2.1.0) with default parameters. The gene abundance was determined using the HTseq software (version 0.5.4p3) in Python to calculate the raw reads number for each gene. The RNAseq data can be obtained from the Gene Expression Omnibus (GEO) database with the accession number GSE70334.

### Differentially Expressed (DE) Genes, Sample Similarity, and Pathways Enrichment

We applied the R package, edgeR (version 3.0.8), for the statistical analysis of the summarized reads. More specifically, the TMM method was used for between-library normalization, and the generalized linear model analysis was used to detect DE genes based on negative binomial models. Genes were declared as DE with the false discovery rate (FDR) controlled at 5% [26] and fold change greater than 1.5. Analysis consisted of the genes with more than 0 cpm in at least 3 samples in each treatment. Principal component analysis (PCA) was applied to the log2 transformed normalized data to examine sample variability of the expression profiles through Qlucore Omics Explorer (v3.0). The DE genes were used to conduct GO and pathway analysis through the GOseq package (version 1.10.0) [27] with FDR controlled at 5%.

### Candidate Gene Validation by qPCR

Ten DE genes identified by RNA-seq were selected for individual qPCR analysis to validate the RNA-seq results. Total RNA was extracted from the same individual samples used in RNA-seq. Primers of the selected genes were obtained from the sequences from NCBI and PRIMER 3 [28], except the primer of pIgR that was from the study of Lammers et al. [29]. The detailed information regarding primers for each selected gene was given in S1 Text. qPCR kits were from Qiagen. The qPCR amplification was carried out with 25 μl reaction mixture including primer, template, QuantiTect SYBR Green RT-PCR Master Mix, QuantiTect RT Mix, and RNase-free water. Cycle conditions were: 30 min at 50°C reverse transcription and 15 min at 95°C PCR initial activation step, then 40 cycles of 15 seconds denaturation at 94°C, 30 seconds annealing at 59°C, and 30 sec extension at 72°C. The housekeeping gene encoding 28S rRNA was used to normalize the start concentration of RNA for the following reasons: (1) 28S rRNA is considered representative of mRNA integrity and its expression tends to be less affected by treatments that significantly alter mRNA expression [30]. (2) 28S rRNA is the most stable gene in different tissues, different development stages, and over a range of temperatures [31]. (3) The qPCR results of our previous studies on APEC showed 28S rRNA was stable in challenged and non-challenged samples by using spleen and leukocytes [24, 25]. Samples from each bird were analyzed in triplicate. The adjusted cycle threshold (Ct) values were obtained by using the following equation: 40 –[Ct target mean + (Ct 28S median–Ct 28S mean) (slope of target/slope of 28S)]. Data were analyzed by JMP statistical software (SAS Institute Inc., Cary, NC). Differences in Ct values between different treatments were used to measure the relative fold changes in gene expression.  

## Results

### Sequencing of Bursal Transcriptomes

**Fig 2:**
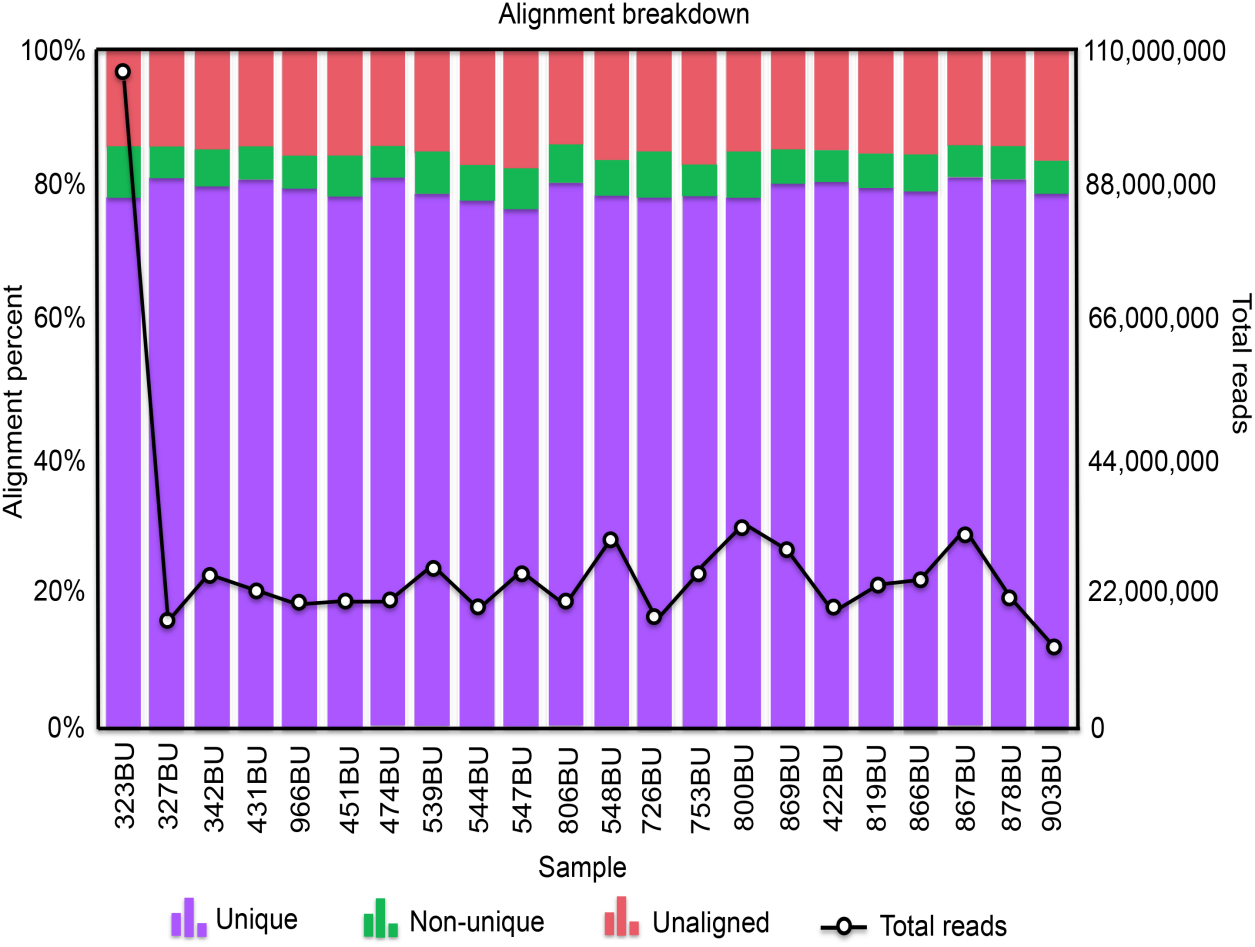
Alignment breakdown plot for all 22 samples. The horizontal axis indicates the individual samples. The left vertical axis and columns show the proportion of reads for each sample with different alignment status. The right vertical axis and the line show the total number of reads for each sample.

**Fig 3:**
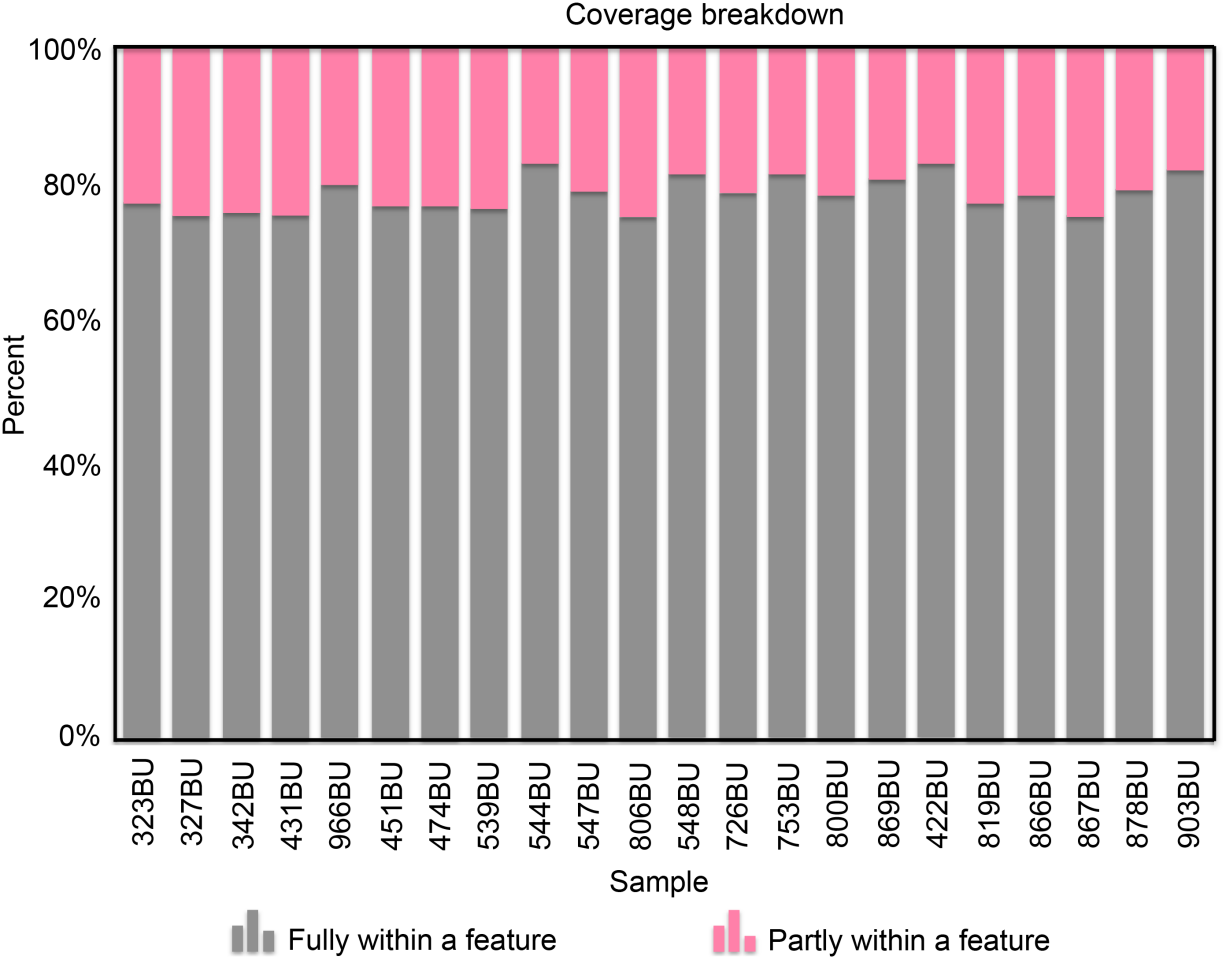
Graphical mapping summary with distribution of mapped reads across the regions of the genome. The x-axis indicates the individual samples and the y-axis shows the percentage of reads mapping to a region of the genome.

To characterize the bursal transcriptomes in response to APEC infection, mRNA of bursa samples were collected from the six treatment groups. After library construction and sequencing, RNAseq produced 14–33 million reads total for 21 libraries. One library had 108 million reads, which may be an artifact resulting from too many products in the PCR amplification step of cDNA library construction, an assumption supported by a high number of duplicate reads. After removing the adaptor and testing quality, an average of 80.35% reads were uniquely mapped to the chicken reference genome Gallus gallus 4 (Fig 2). The coverage is approximately 80% per library for reads fully within a gene and about 20% for reads partly within a gene (Fig 3). A total of 16,603 genes were detected and 2,469 of those were novel genes. After the removal of genes with low reads (genes with 0 read counts in at least three samples in each treatment group), 11,169 genes were included for further statistical analysis.

### Sample Variability and Detected DE Genes

**Fig 4:**
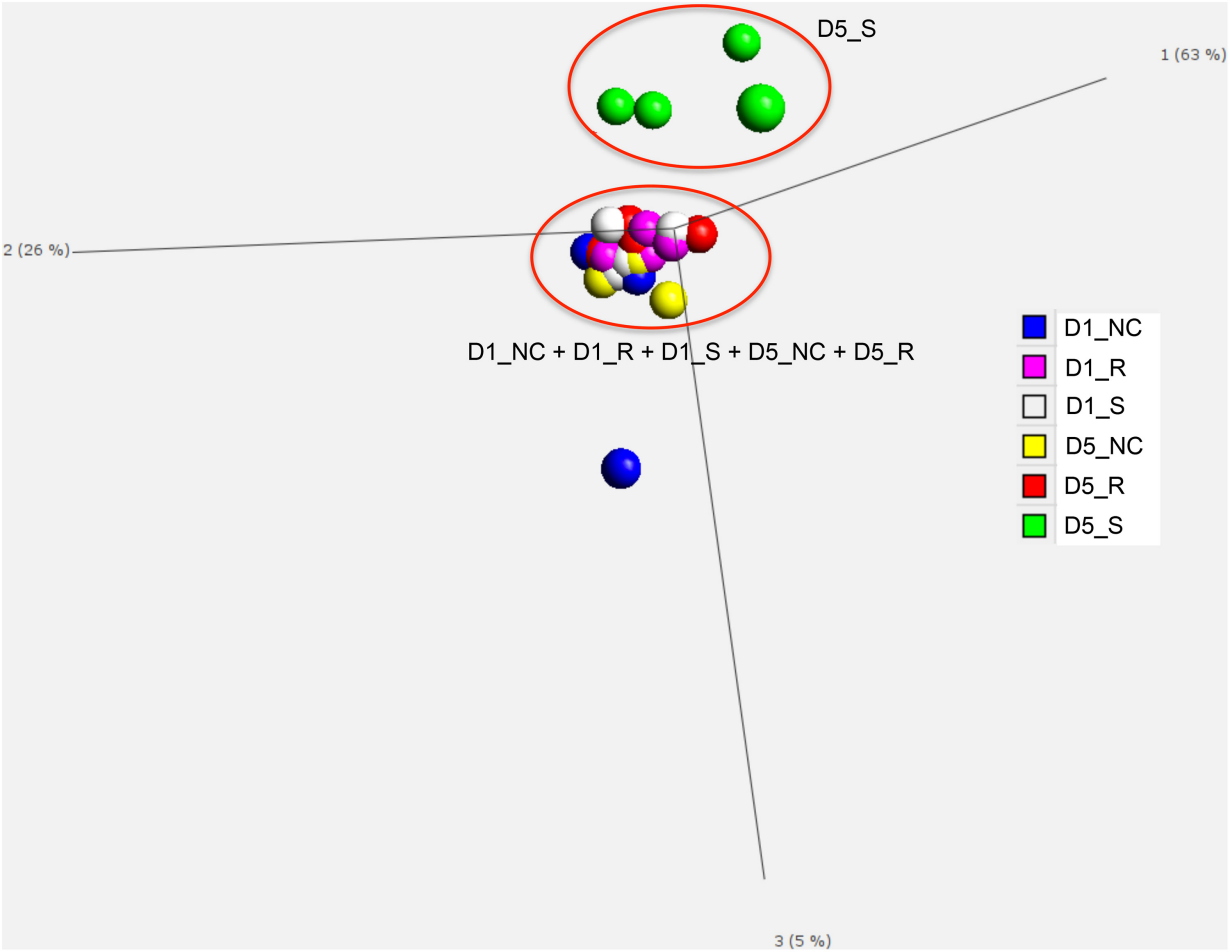
Principle component analysis (PCA). The PCA scatter plot of all 22 samples was generated by using Qlucore Omics Explorer v3.0 to evaluate the variability of RNAseq data. The first principle component accounted for 63% of the total variation in the data. The second and third principle components represented 26% and 5% of the overall variation in the data, respectively. D5_S, susceptible birds at 5 days post-infection (dpi); D5_R, resistant birds at 5 dpi; D5_NC, non-challenged birds at 5 dpi; D1_S, susceptible birds at 1 dpi; D1_R, resistant birds at 1 dpi; D1_NC, non-challenged birds at 1 dpi.

To visualize the pattern of relatedness of the treatment groups, all samples were subjected to PCA (Fig 4). There was a clear separation of challenged-susceptible birds at 5 dpi from the other five treatment groups. The resistant and the non-challenged birds at 1 or 5 dpi, and the susceptible birds at 1 dpi, were tightly clustered, except for one outlier, suggesting that there was a very similar gene expression pattern between the resistant and non-challenged birds, as well as the susceptible birds at 1 dpi.

Nine two-way contrasts were generated based on the detectible effects of pathology and time post-infection factors (Table 2). Large numbers of DE genes were enriched in three contrasts: susceptible vs. non-challenged birds at 5 dpi, susceptible vs. resistant birds at 5 dpi, and 5 dpi vs. 1 dpi in susceptible birds (Table 2); the other contrasts had few DE genes (N<25). These results indicate the unique nature of the challenged-susceptible birds’ response at 5 dpi. There was little difference in the bursal transcriptome between challenged-resistant and non-challenged birds on either 1 or 5 dpi. Moreover, the transcriptomes of susceptible, resistant, and non-challenged birds at 1 dpi differed very little. There was also little effect of time post-infection in challenged-resistant and non-challenged birds.

**Table 2:** Numbers of Significantly Differentially Expressed (DE) Genes with FDR<5% & FC>1.5.

Treatment contrast	# of DE genes	# of ↑ DE genes	# of ↓ DE genes
Susceptible vs. Non-challenged birds at 1 dpi	25	6	19
Resistant vs. Non-challenged birds at 1 dpi	2	0	2
Susceptible vs. Resistant birds at 1 dpi	2	2	0
Susceptible vs. Non-challenged birds at 5 dpi	3165	1743	1422
Resistant vs. Non-challenged birds at 5 dpi	1	1	0
Susceptible vs. Resistant birds at 5 dpi	748	456	292
5 dpi vs. 1 dpi in susceptible birds	99	91	8
5 dpi vs. 1 dpi in resistant birds	1	0	1
5 dpi vs. 1 dpi in non-challenged birds	2	2	0

Note: dpi, day post-infection, #, number.

**Fig 5:**
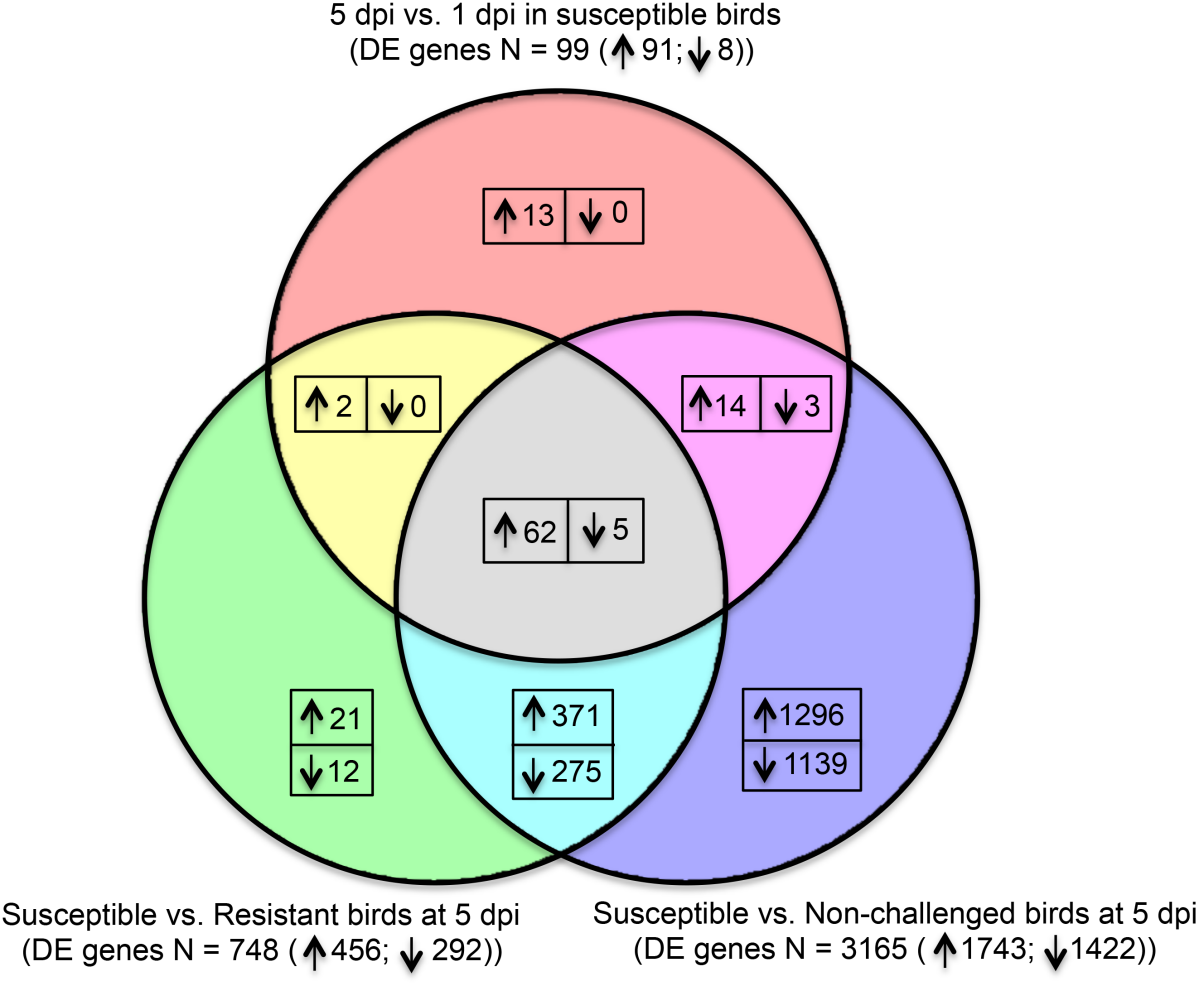
Shared and unique significantly differentially expressed (DE) genes in the three contrasts of susceptible vs. non-challenged birds at 5 days post-infection (dpi), of susceptible vs. resistant birds at 5 dpi, and of 5 dpi vs. 1 dpi susceptible birds. ↑, up-regulated; ↓, down-regulated; N, number.

Fig 5 shows the shared and unique DE genes of the three contrasts, susceptible vs. non-challenged birds at 5 dpi, susceptible vs. resistant birds at 5 dpi, and 5 dpi vs. 1 dpi in susceptible birds. Sixty-seven DE genes were co-expressed in the three contrasts, including 62 up-regulated genes and 5 down-regulated genes. The concordance of the direction of expression of the shared DE was 100% in the three contrasts. A total of 646 DE genes (371 up-regulated and 275 down-regulated) were shared in expression in susceptible vs. non-challenged birds at 5 dpi and susceptible vs. resistant birds at 5 dpi. And a total of 2435 DE genes (1296 up-regulated and 1139 down-regulated) were uniquely expressed in susceptible vs. non-challenged birds at 5 dpi.

### GO Term Assignment

GO analysis was conducted to identify the main biological processes in which DE genes from the three major contrasts were involved: susceptible vs. non-infected birds at 5 dpi, susceptible vs. resistant birds at 5 dpi, and susceptible birds at 5 vs. 1 dpi. Overall, similar significant GO terms were detected in the contrast of susceptible vs. non-challenged birds at 5 dpi and of susceptible vs. resistant birds at 5 dpi. These GO terms included regulation of cell proliferation, cellular response to stress, cell cycle, DNA replication, and phosphate metabolic process. However, many immune response significant GO terms were involved in the contrast of 5 dpi vs. 1 dpi in susceptible birds. These GO terms were positive regulation of leukocyte activation, positive regulation of cell activation, positive regulation of immune system process, defense response to bacterium, and leukocyte differentiation.

### Analysis of Significantly Changed Pathways

The up- and down-regulated DE genes in the contrast of susceptible vs. non-challenged birds at 5 dpi, of susceptible vs. resistant birds at 5 dpi, and of 5 dpi vs. 1 dpi in susceptible birds were separately analyzed to detect the significant pathways, with FDR controlled at 5%. For the up-regulated DE genes, four pathways were significantly changed for the contrast of susceptible vs. non-challenged birds at 5 dpi and three of the four significant pathways were identified in susceptible vs. resistant birds at 5 dpi (Table 3). The four significantly enriched pathways were mainly related to defense mechanism and signal transduction: cytokine-cytokine receptor interaction, lysosome, cell adhesion molecules (CAM), and apoptosis (Table 3). Except the CAM pathway, the up-regulated DE genes in the contrast of 5 dpi vs. 1 dpi in susceptible birds had specific enriched pathway, transforming growth factor-beta (TGF-beta) signaling (Table 3). For down-regulated DE genes, three significant pathways, cell cycle, B cell receptor signaling, and p53 signaling pathway, were identified in both susceptible vs. non-challenged birds at 5 dpi and susceptible vs. resistant birds at 5 dpi (Table 3).

**Table 3:** Significantly Changed Pathways in Different Contrasts.

Contrast	Up-regulated DE genes		Down-regulated DE genes	
	Pathway name	Adjusted p value	Pathway name	Adjusted p value
Susceptible vs. Non-challenged birds at 5 dpi	gga04060: Cytokine-cytokine receptor interaction	1.41E-05	gga04110: Cell cycle	5.19E-11
	gga04142: Lysosome	1.63E-03	gga04662: B cell receptor signaling pathway	1.62E-03
	gga04514: Cell adhesion molecules (CAMs)	3.52E-03	gga04115: p53 signaling pathway	4.93E-03
	gga04210: Apoptosis	3.64E-02		
Susceptible vs. Resistant birds at 5 dpi	gga04142: Lysosome	6.52E-03	gga04110: Cell cycle	8.70E-11
	gga04514: Cell adhesion molecules (CAMs)	1.71E-02	gga04115: p53 signaling pathway	1.93E-03
	gga04060: Cytokine-cytokine receptor interaction	2.46E-02	gga04662: B cell receptor signaling pathway	8.27E-03
5 dpi vs. 1 dpi susceptible birds	gga04514: Cell adhesion molecules (CAMs)	1.35E-04		
	gga04350: TGF-beta signaling pathway	4.40E-03		

Note: DE, differentially expressed.

### qPCR data analysis

The qPCR assays were conducted to validate eleven selected DE genes from RNAseq, which were BLNK, BTK, CASP3, CASP10, CD28, IFNG, CD3Z, ZAP70, LCK, FAS, and PIGR. Strong correlation was observed between qPCR and RNAseq results for these genes. Pearson’s correlation of the fold changes between qPCR and RNAseq was 0.95. While controlling the type I error rate at 5% (p-value < 0.05), the qPCR results indicated that the expression of all the 11 genes was consistent in significance and fold changes with that shown by RNAseq (Table 4).

**Table 4:** Quantitative PCR Validation.

Gene	Contrast	qPCR	RNA-seq
BLNK	Susceptible vs. Non-challenged birds at 5 dpi	-2.83**	-2.30**
	Susceptible vs. Resistant birds at 5 dpi	-2.54**	-2.00**
BTK	Susceptible vs. Non-challenged birds at 5 dpi	-2.07**	-2.10**
	Susceptible vs. Resistant birds at 5 dpi	-2.81**	-1.71**
	5 dpi vs. 1 dpi susceptible birds	-3.93**	-1.89**
CASP3	Susceptible vs. Resistant birds at 5 dpi	+2.22*	+1.97**
CASP10	Susceptible vs. Non-challenged birds at 5 dpi	+2.17**	+2.85**
CD28	Susceptible vs. Non-challenged birds at 5 dpi	+21.95**	+27.81**
	Susceptible vs. Resistant birds at 5 dpi	+18.53**	+16.70**
IFNG	Susceptible vs. Non-challenged birds at 5 dpi	+3.53*	+2.81**
	Susceptible vs. Resistant birds at 5 dpi	+4.68**	+4.79**
CD3Z	Susceptible vs. Non-challenged birds at 5 dpi	+3.29**	+3.55**
	Susceptible vs. Resistant birds at 5 dpi	+3.14*	+3.91**
	5 dpi vs. 1 dpi susceptible birds	+5.28**	+4.57**
ZAP70	Susceptible vs. Non-challenged birds at 5 dpi	+5.92*	+7.97**
	Susceptible vs. Resistant birds at 5 dpi	+7.86**	+11.07**
	5 dpi vs. 1 dpi susceptible birds	+6.43**	+9.28**
LCK	Susceptible vs. Non-challenged birds at 5 dpi	+4.80**	+7.63**
	Susceptible vs. Resistant birds at 5 dpi	+5.47**	+8.78**
	5 dpi vs. 1 dpi susceptible birds	+7.68**	+8.97**
FAS	Susceptible vs. Non-challenged birds at 5 dpi	+2.58**	+1.74**
PIGR	Susceptible vs. Non-challenged birds at 5 dpi	-2.96**	-4.58**

Note: Fold change between contrasts presented in third and fourth column. + values indicate higher expression in the first group,—values indicate higher expression in the second group.

** P value < 0.01,

* P value < 0.05 in qPCR and RNA-seq. dpi, days post-infection.

## Discussion

This novel experiment characterized changes in the chicken bursal transcriptome associated with two extreme pathology (lesion) levels in response to APEC infection, as well as differences between non-infected and challenged birds. The large number of infected birds (N = 288) enabled us to identify a sufficient range of lesion scores to separate distinct pathology groups. The total lesion score distribution for infected birds reported in previous published papers [24]. At 1 dpi, the total lesion score distribution was nearly normal, with few extreme birds [24]. At 5 dpi, however, the distribution became right skewed [24] as the number of high total lesion birds increased significantly.

Classification of resistant and susceptible birds by lesion scores was confirmed by the PCA of the transcriptome results in which the 5 dpi susceptible birds were distinct from the resistant and non-challenged birds. Few DE genes were detected in the contrasts at 1 dpi (Table 1), which is consistent with the PCA results. These results suggest that bursal cells are mainly involved in the adaptive immune response, which is not activated as early as 1 dpi. Therefore, we detected little difference at 1 dpi among susceptible, resistant, and non-challenged birds.

Three contrasts generated large numbers of DE genes (N > 25): susceptible vs. non-challenged birds at 5 dpi, susceptible vs. resistant birds at 5 dpi, and 5 dpi vs. 1 dpi susceptible birds (Table 2). The 62 shared, up-regulated DE genes of these three contrasts were mainly related to immune function, such as lymphocyte cytosolic protein 2 (SLP76), IL2-inducible T-cell kinase (ITK), lymphoid enhancer-binding factor 1 (LEF-1), and immunoglobulin-like domain containing receptor 1 (ILDR1) (data in S2 Text). The five down-regulated DE genes of the three contrasts were tyrosine-protein kinase (BTK), squalene synthase (FDFT1), hemopexin (HPX), and two novel genes. These findings differ from the results of spleen and caecum after Salmonella infection [32, 33], although many immune genes were also detected in Matulova et al. study. In the Matulova et al., [32, 33] study, the discovered immune genes were IgG, IRG1, IL-22, IFNγ, iNOS, IL-1β, which significantly changed under Salmonella infection in spleen and caecum. The different results between the Matulova et al. and the current study may have arisen because of the differences in the tissues studied, the bacterium used for challenge, and the genetics of the chickens.

APEC has several infection routes. The main route of infection is inhalation of contaminated dust through the respiratory system [34]. From the respiratory tract, APEC can quickly enter into the bloodstream and colonize the internal organs including lung, air sac, and liver [35]. Based on lesion scores (Table 1), it was clear that septic infection occurred in the challenged birds. Colony counts were not performed on all tissues, so the bursa itself may not have been colonized in all birds, however, many studies report local involvement of the bursa in colibacillosis [20, 21].

The significantly changed pathways detected for the three major contrasts were in strong agreement with our hypothesis of alteration in B cell proliferation and differentiation. The B cell receptor (BCR) signaling and cell cycle were significantly suppressed in susceptible vs. non-challenged birds at 5 dpi and susceptible vs. resistant birds at 5 dpi (Table 3). The BCR signaling pathway is essential to trigger orchestrated intracellular signaling cascades, resulting in B cell proliferation, differentiation, survival and activation [36, 37]. The BCR is a heterotrimeric complex including Ig α (CD79A) and Ig β (CD79B) whose tail immunoreceptor tyrosine-based activation motif (ITAM) is essential to signal transduction [38, 39]. The ITAM is phosphorylated by Src-family kinase (SFK), LYN, and binds to kinase SYK [40–42]. After SYK activation, the adaptor protein B-cell linker (BLNK) is phosphorylated and serves as a scaffold to assemble other components: Bruton’s tyrosine kinase (BTK) and VAV [42–44]. The VAV and BTK can further lead to RAC and PLC γ pathway, respectively.

In the current study, genes CD79B, BTK, BLNK, BLK, VAV were all significantly down-regulated in susceptible vs. non-challenged birds at 5 dpi. And, importantly, three of these genes (BTK, BLNK, and BLK) were also identified as differently expressed in susceptible vs. resistant birds at 5 dpi. These results, together with the known involvement of the bursa and immunoglobulins in defense against colibacillosis [17, 21], suggest that the BCR signaling pathway is an important mechanism in response to APEC-induced pathogenesis. Detailed information on the DE genes was displayed in in S3 Text.

Although differences in immunoglobulins (IgA, IgM, and IgY) were not detected, the expression of the PIGR gene was decreased in susceptible vs. non-challenged birds at 5 dpi. This gene was also validated to be significantly changed by using qPCR (Table 4). The PIGR gene encodes a polymeric Ig receptor that is a key component of secreted IgA [45]. Because PIGR binds to conserved areas of IgA, it does not depend on antigen specificity [45]. Increasing expression of PIGR in the bursa might be more important than upregulating the total number of Ig class genes because the receptor does not require any additional specificity. If Ig class gene specificity increases outside the bursa, then more receptors will be needed for Ig transcytosis. Thus, the decreased expression of PIGR in the bursa of susceptible birds may indicate a defective mechanism in response to APEC.

Many defense pathways were significantly induced in susceptible vs. non-challenged birds at 5 dpi, including cytokine-cytokine receptor interaction, lysosome, CAM, and apoptosis pathways (Table 3). The lysosome, CAM and apoptosis pathways were previously identified in leukocytes of APEC-infected susceptible birds compared to non-challenged birds at 5 dpi [25]. The lysosome, CAM, and cytokine-cytokine receptor interaction pathway were also significantly changed in susceptible vs. resistant birds at 5 dpi (Table 3). Thus, the lysosome and apoptosis are implicated as two common pathways of response to APEC in susceptible birds. The significantly changed cytokine-cytokine receptor interaction pathway suggests that these soluble factors are a mechanism of signaling to or by the bursa to enable systemic effects in response to APEC sepsis. The DE genes involved in each of the before mentioned significant pathways were described in S3 Text.

Apoptosis, a major cell death process, plays an essential role in organismal growth and tissue homeostasis [46, 47]. Cell apoptosis has been associated with APEC infection in many studies: Gao et al. [48] found that APEC-induced apoptosis occurred in chicken embryo intestinal cells; Horn et al. [49] and Bastiani et al. [50] determined that APEC infection induces macrophages apoptosis; Sandford et al. [25] reported changes in apoptosis-related genes in the challenged-susceptible birds at 5 dpi. The extrinsic apoptosis pathway is initiated by the activation of death receptor FAS, a member of the tumor necrosis factor (TNF) receptor superfamily [51]. Then, FAS binds to CASP8 or CASP10, leading to the activation of the pro-apoptotic proteins BID and BAX through tumor suppressor protein p53 [52]. Moreover, Wei et al. demonstrated the JNK pathway was initiated during the BAX-mediated apoptosis responses [53]. In the contrast of susceptible vs. non-infected birds at 5 dpi in the current study, 15 up-regulated DE genes with fold changes of 1.5 to 9.0 participated in the apoptosis pathway, including FAS, IL1R, CASP3, CASP10, and CFLAR, (S3 Table). The significant pathways, CAM and TGF-beta signaling pathway, were also detected. The TGF-beta signaling pathway has an important role in cell growth, cell differentiation, apoptosis, and cellular homeostasis [54]. The CAM is critical to development, maintenance of homeostasis, immune and inflammatory responses, tissue repair, cell migration, and apoptosis [55–58]. Increased expression of many genes in the apoptosis pathway in susceptible birds strongly suggests the importance of this mechanism in the previously reported phenomenon of bursal atrophy that accompanies APEC infection [20, 21].

Resistant birds appear to activate the p53 signaling pathway in response to APEC infection. The p53 protein, known as a major tumor suppressor, functions to inhibit cell proliferation programs through apoptotic cell death and cell cycle arrest [59–61]. Moreover, p53 can also accelerate cell differentiation and DNA repair [62]. The CDK1, CCNB2, and CCNB3 involved in p53 signaling pathway have important function in inhibiting cell proliferation (G2/M transition) and accelerating DNA repair [63, 64]. In the current study, total five DE genes (CDK1, CCNB2, CCNB3, CCNE2, and CYC) participated in the activated p53 signaling pathway in resistant birds (data in S3 Text). These results suggest that the APEC-infected, resistant birds increase cell differentiation in the bursa, instead of cell proliferation.

In summary, the current study is the first to characterize the transcriptomic responses of the bursa, the avian-specific developmental tissue source of B cells, to APEC infection. The DE genes involved in the BCR signaling pathway are strong candidates for markers for resistant birds. The challenged-susceptible birds exhibited strong suppression of the BCR signaling pathway, which may be a major defect causing susceptibility to APEC-induced pathology. Challenged-susceptible birds also showed induction of many defense pathways, including apoptosis and lysosome, which may be common pathways for susceptible birds in response to APEC infection and pathology. The TGF-beta signaling and CAM pathway may function in local tissue repair in the bursa. This study provides considerable insight into potential mechanisms of resistance and susceptibility to ExPEC infection, thus paving the way to develop strategies for ExPEC prevention and treatment, as well as enhancing innate resistance by genetic selection in animals.

## Supporting Information

S1 Table Primers sequence for qPCR validation. (XLSX)

S2 Table Shared and unique differentially expressed genes in the contrast of susceptible vs. non-challenged birds at day 5, of susceptible vs. resistant birds at day 5, and of day 5 vs. day 1 susceptible birds. Legends: NA, not available; S5, day 5 susceptible birds; NC5, day 5 non-challenged birds; R5, day 5 resistant birds; S1, day 1 susceptible birds.
(XLSX)

S3 Table The differentially expressed genes in each of the significantly changed pathways in the three contrasts: susceptible vs. non-challenged birds at day 5, susceptible vs. resistant birds at day 5, and day 5 vs. day 1 susceptible birds. (XLSX)
